# SpecieScan: semi-automated taxonomic identification of bone collagen peptides from MALDI-ToF-MS

**DOI:** 10.1093/bioinformatics/btae054

**Published:** 2024-02-09

**Authors:** Emese I Végh, Katerina Douka

**Affiliations:** Department of Evolutionary Anthropology, University of Vienna, University Biology Building, A-1030 Vienna, Austria; Human Evolution and Archaeological Sciences (HEAS), University of Vienna, Vienna, Austria; Archaeology, Environmental Changes, and Geochemistry, Vrije Universiteit Brussel, 1050 Brussels, Belgium; Department of Evolutionary Anthropology, University of Vienna, University Biology Building, A-1030 Vienna, Austria; Human Evolution and Archaeological Sciences (HEAS), University of Vienna, Vienna, Austria

## Abstract

**Motivation:**

Zooarchaeology by Mass Spectrometry (ZooMS) is a palaeoproteomics method for the taxonomic determination of collagen, which traditionally involves challenging manual spectra analysis with limitations in quantitative results. As the ZooMS reference database expands, a faster and reproducible identification tool is necessary. Here we present SpecieScan, an open-access algorithm for automating taxa identification from raw MALDI-ToF mass spectrometry (MS) data.

**Results:**

SpecieScan was developed using R (pre-processing) and Python (automation). The algorithm’s output includes identified peptide markers, closest matching taxonomic group (taxon, family, order), correlation scores with the reference databases, and contaminant peaks present in the spectra. Testing on original MS data from bones discovered at Palaeothic archaeological sites, including Denisova Cave in Russia, as well as using publicly-available, externally produced data, we achieved >90% accuracy at the genus-level and ∼92% accuracy at the family-level for mammalian bone collagen previously analysed manually.

**Availability and implementation:**

The SpecieScan algorithm, along with the raw data used in testing, results, reference database, and common contaminants lists are freely available on Github (https://github.com/mesve/SpecieScan).

## Graphical Abstract

**Graphical Abstract:**
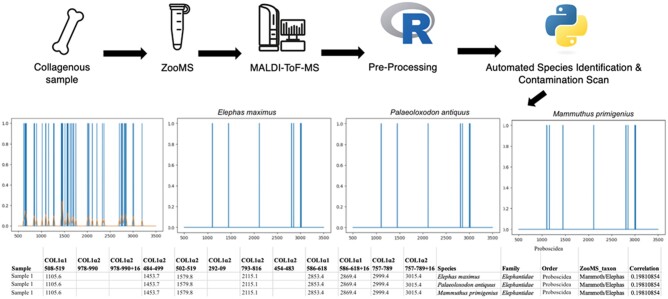

## 1 Introduction

Matrix-assisted laser desorption/ionization (MALDI) time-of-flight (TOF) mass spectrometry (MS) has gained popularity in the past decades in the analysis of a range of biological molecules and biopolymers. When used on collagen, peptide mass fingerprinting through MALDI-ToF-MS is a powerful technique for the taxonomic determination of organisms; the method is now known to archaeologists and anthropologists as zooarchaeology by mass spectrometry (ZooMS) (Buckley *et al.* 2009).

ZooMS can deliver robust taxonomic identifications of archaeological tissues (for a recent review see: Richter *et al.* 2022) and has been applied to a range of collagenous material, such as parchment (Kirby *et al.* 2013, Teasdale *et al.* 2017), antler (von Holstein *et al.* 2014), ivory (Coutu *et al.* 2016), and leather (Ebsen *et al.* 2019). However, the method has had major impact on the identification of morphologically non-diagnostic and/or fragmented bone from prehistoric sites (Brown *et al.* 2016, 2022, Welker *et al.* 2016).

Although DNA-based methods have a larger taxonomic resolution than peptide mass fingerprinting, ZooMS is often used for screening a large number of bones in a cost-, resource-, and time-effective way (Gu & Buckley 2018). Moreover, since bone collagen and other proteins are known to survive longer than DNA and RNA (Buckley *et al.* 2008), ZooMS can be applied to samples from deep time, to further understand biodiversity, human–animal interactions, as well as cost-effectively scanning large amount of bone fragments for human remains (Douka *et al.* 2019).

The level of taxonomic specificity reached by ZooMS varies and is determined by the evolutionary differences and split times between the groups being classified. The choice of peptide markers plays a crucial role in determining the level of taxonomic resolution. ZooMS can typically distinguish between family and in some cases subfamily level (e.g. cetaceans; Evans *et al.* 2016), or even genus level for sheep/goat (Buckley *et al.* 2010), some bovids (Janzen *et al.* 2021), and cervids (Jensen *et al.* 2021).

Taxonomic identifications (IDs/ZooMS taxon) are normally established by the careful manual examination of mass spectrometric data. In this process, specific peptide ‘marker’ peaks act as a barcode, allowing for the association of particular peaks (or a combination thereof) with a specific taxon present in a reference database containing these markers. Occasionally, the peptide markers in an unknown sample may differ from those of the same species in the reference database, due to e.g. mass shifts caused by post-translational modifications (PTMs) in one or two peptide markers (Hickinbotham *et al.* 2020). The presence of PTMs in archaeological bone proteins can be attributed to various factors, e.g. natural occurrence during the organism’s lifetime, as part of the normal processes of protein synthesis and metabolism. Common *in vivo* PTMs in collagen include the hydroxylation of proline and lysine residues (Creecy *et al.* 2021) or the addition of phosphoryl or carbohydrate groups (Hendy 2021). PTMs may also occur after the organism’s death and the subsequent burial of bone tissues. Factors such as enzymatic activity by microorganisms or chemical degradation of bone tissue may lead to protein modifications (Schroeter and Cleland 2016, Hendy 2021). Additionally, PTMs may be introduced during sample preparation and analysis. For example, certain chemicals or conditions during laboratory preparation can lead to phenomena such as glutamine deamidation of collagen peptides (Simpson *et al.* 2016).

Due to these issues, taxonomic determination from raw MALDI-ToF-MS data is not straightforward and requires training and expert judgement. Even for experienced users, manual data analysis is time and effort intensive. A routine workflow includes visual inspections of mass spectra (in our lab done using the open-access software mMass; Niedermeyer and Strohalm 2012; https://github.com/xxao/mMass) that was recently updated by our FINDER project here: https://github.com/dreamingspires/mMass. Available online training material (e.g. https://www.protocols.io/view/identifying-zooms-spectra-mammals-using-mmass-kqdg36rppg25/v1) sets a standard process of peptide markers to be analysed in an order: COL1ɑ2 484–498 (B) > COL1ɑ2 793–816 (D) > COL1ɑ2 978 –990 (A) > COL1ɑ2 502–519 (C) > COL1ɑ2 454–483 (E) > COL1ɑ1 586–618 (F) > COL1ɑ2 757–789 (G) > COL1ɑ1 508–519 (P1) > COL1ɑ2 292–309 (P2). Manual MS data analysis using this order of peptide markers together with the available reference database (https://www.york.ac.uk/archaeology/centres-facilities/bioarch/facilities/zooms/) could sometimes produce results of questionable reliability. The omission of one of the first single peptide markers in this order can jeopardize the subsequent analyses. In variably degraded samples, the ‘true’ peak marker of COL1ɑ2 793–816 is occasionally absent, while adjacent peaks might be present. Consequently, the only way to overcome this issue is a manual re-examination of the spectra using different orders of peptide markers and noting down all possible masses for each peptide peak marker. Furthermore, taxonomic identifications produced this way might incorporate species inappropriate for the geographical context in question. Thus, another manual step is necessary to curate and remove these geographically irrelevant identifications from the results. These complications make the manual data analysis even more time and effort intensive.

The present study aims at automating the taxonomic identification process of MALDI-ToF mass spectra. Ultimately, this will enhance (i) efficiency—the automation of the data analysis process can significantly reduce the time and effort required to analyse large amounts of data. This can be particularly useful in high-throughput ZooMS analysis, where researchers may need to analyse thousands of spectra in a single study; (ii) accuracy—automated data analysis can be more accurate than manual analysis, as it is less prone to human error and it does not have to follow a standard order of peptide markers for analysis; (iii) consistency—automated data analysis ensures that the same methods and criteria are used to analyse all data, ensuring that the results are produced using consistent methodologies; (iv) reproducibility—automated data analysis ensures that the results of an experiment/study are reproducible; and, finally, (v) future scope—when new species are added to the reference database and increase the taxonomic resolution for a geographical area, previously analysed MALDI-ToF-MS data can be easily rerun to offer more accurate IDs.

So far, two algorithms have been developed for the automated taxonomic identification of MALDI-ToF-MS data for ZooMS (Gu and Buckley 2018, Hickinbotham *et al.* 2020). Hickinbotham *et al.* (2020) introduced an open-access R-based algorithm tailored to the identification of collagen from parchment, originally designed for three species (*Ovis*, *Bos*, *Capra* sp.). Although it serves its purpose effectively, there are some limitations to accommodate a wider range of species and materials, as shown by the scope of its use to date on parchments and palimpsests (Ruffini-Ronzani *et al.* 2021, Viñas-Caron *et al.* 2023). Gu and Buckley (2018) suggested a semi-supervised machine learning algorithm developed for five species, but the algorith is not openly available. Our newly introduced algorithm, SpecieScan, uses all taxa from the online available reference database, it is open-access, does not require intensive computational skills, and is easy to use with little prior training.

## 2 Materials and methods

Three types of data were used in this study: (i) data generated by one of us (E.V.) in-house at the Palaeoproteomics Laboratory (Douka Lab), Department of Evolutionary Anthropology, University of Vienna; (ii) data generated by a different user at the same laboratory; and (iii) data acquired from open-access supplementary materials from published, peer-reviewed papers. It was necessary to include data produced by other laboratories in order to test the applicability of the algorithm to raw MALDI-Tof mass data obtained by other instruments.

### 2.1 Materials

The bone samples prepared and analysed in-house by E.V. come from the Denisova Cave, Russia (*N* = 290). These samples were prepared at the Palaeoproteomics Labs (Douka Lab), University of Vienna, between 2022 and 2023. Other internal samples included in the testing of the algorithm come from Vogelherd Cave, Germany (*n* = 267, Bloß 2023). The taxonomic identifications from Vogelherd were originally generated by a different internal software (QuickID, developed by Wang and Douka, unpublished work), and subsequently the identifications were confirmed manually by Bloß (2023). All internally prepared samples are of Palaeolithic age.

Externally analysed collagen used for testing the accuracy of the algorithm comes from six archaeological sites (Kapiri Mposhi A, Fibobe II, Muteteshi, Kalundu Mound on the Batoka Plateau, Salumano, and Jakobo West on the margin of the Kalahari Depression) in Zambia, Africa (Janzen *et al.* 2021), Bandicoot Bay, Barrow Island, Australia (Peters *et al.* 2021), and Bardha’a, Azerbaijan (Wordsworth *et al.* 2021). The raw mass spectra and the taxonomic identifications associated with these published studies were downloaded from the online repository Zenodo.

### 2.2 Peptide mass fingerprinting

Bone chips (15–25 mg) were removed from each bone fragment after surface cleaning with AlO_2_ powder. All internal subsampled faunal bone fragments from Denisova Cave underwent collagen extraction following the acid-insoluble extraction protocol (Welker *et al.* 2015). Briefly, 500 μl of cold (4°C) 0.6 M HCl was added to each bone chip and the sample was left to demineralize in the fridge (4°C) for 1–2 days. The acid supernatant was removed, and the samples were rinsed 3 times with 200 μl of 50 mM pH 8 ammonium biocarbonate (NH_4_HCO_3_; AmBic). Humic acids were removed by adding 200 μl of 0.1 M NaOH, followed by 200 μl of 50 mM AmBic (pH 8) three times, which brought the solution back to neutral pH. Subsequently, 100 μl of AmBic was added to each sample followed by incubation in an oven for 1 h at 65°C. After centrifugation, 50 μl of the resulting supernatant was incubated with 1 μl of trypsin solution (0.4 μl/μg) (ThermoFisher Pierce™ Trypsin Protease) at 37°C for 12–18 h. Following tryptic digestion, the samples were centrifuged and were acidified to 1 μl of 5% trifuoroacetic acid (TFA) and the peptides were purified and extracted with a 100 μl C18 resin ZipTip^®^ pipette tip with conditioning solution, composed of 0.1% TFA in 50% acetonitrile, and washing buffer solution, composed of 0.1% TFA in ultrapure MilliQ™ water. Samples were eluted with 50 μl conditioning solution.

Bruker peptide calibrant standards were spotted (0.5 μl/spot) on a ground steel 385-spot MALDI target plate along with diluted (1:10) tryptic collagen peptides in 0.5 μl/spot triplicates. Samples were spotted on ground-steel MALDI plates in triplicates mixed with 0.5 μl of α-cyano-4-hydroxycinnamic acid matrix solution. Analyses were run on an in-house Bruker Autoflex Speed MALDI-ToF-MS (Bruker Daltonics).

The generated spectra were first visually inspected using the mMass software (Niedermeyer and Strohalm 2012), where possible, using the established order of peptide markers. The technical replicates were not merged for manual analysis; instead, the three replicates were inspected simultaneously. The peptide markers were analysed settings: baseline correction (precision: 15, relative offset: 25), Savitzky–Golay smoothing (window size: offset: 0.3 *m/z*, cycles: 2, SNR = 3.0), and their atomic mass values (in Da) were recorded with the order, family, and species information prior to comparison with the results of the algorithm.

### 2.3 Reference databases

The reference database used in this study was the open-access dynamic peptide marker database available at: https://www.york.ac.uk/archaeology/centres-facilities/bioarch/facilities/zooms/. The downloaded data was cleaned using the Pandas library in Python programming language. This included, but was not limited to, removing inconsistencies in punctuations, creating a new row for peptide markers that had more entries for the same species, as well as adding a new column ‘ZooMS_taxon’. Species were separated based on their respective geographical areas and saved as .csv files, creating a separate reference database for mammals from Eurasia, Africa, and the New World. Species separation by geographical areas into their own .csv files was necessary, because family members of Eurasian and African taxa often share multiple peptide markers, which resulted in geographically imprecise results when identifying species manually and when testing the algorithm. Although Eurasian and African species can indeed exhibit shared peptide markers, the notion of using a singular database for all geographical areas would require additional analyst input to exclude geographically irrelevant taxa. Hence, the decision to compartmentalize the data into separate .csv files based on geographical regions serves to streamline the identification process, enhance accuracy, and mitigate challenges posed by shared peptide markers. Reptiles, birds, amphibians, and fish were not the focus of this study, but the cleaned reference databases are available in the supplementary materials on GitHub.

### 2.3 Algorithm

#### 2.3.1 Data pre-processing

The data pre-processing was carried out in the R programming language using the MALDIquant, MALDIquantForeign (Gibb and Strimmer 2012), and factoextra packages, as adapted from Codlin *et al.* (2022), and subsequently modified and further developed prior to automation in Python. The R code and the associated ‘README’ file used in this pre-processing workflow are available on GitHub (https://github.com/mesve/SpecieScan).

**Figure 1.**
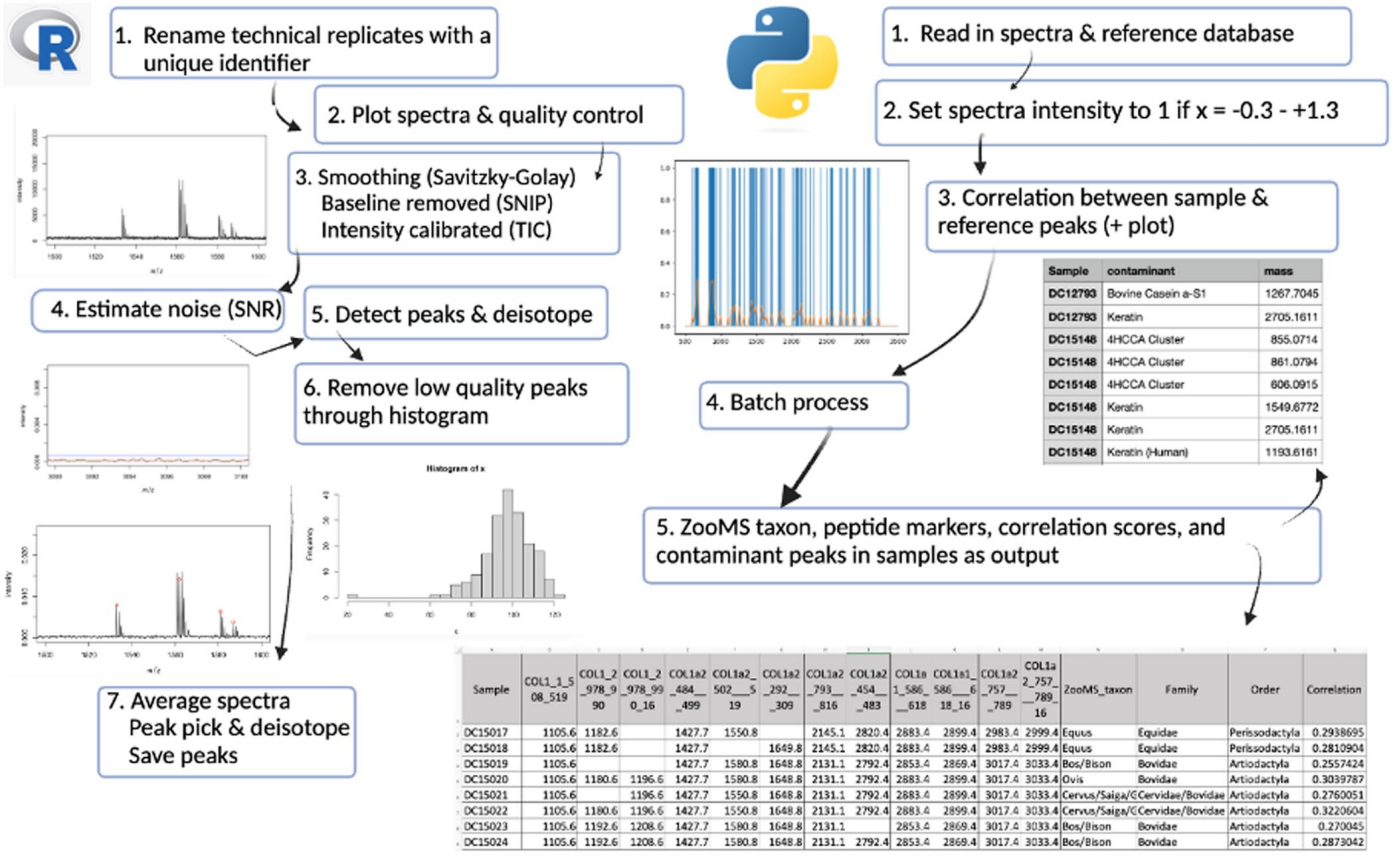
SpecieScan workflow schematic showing the steps involved in the R and Python algorithms for the taxonomic determination of collagen from raw MALDI-ToF-MS data.

Figure 1 illustrates a schematic workflow of the pre-processing steps in R and the automated taxonomic identification steps in Python. The pre-processing steps involve the visualization and quality control of the imported raw data to ensure the absence of empty elements in the spectra objects and to verify the regularity of their lengths. Next, the Savitzky–Golay smoothing function is applied to the spectra to remove noise and to smooth peaks or valleys in the spectral data, using the smoothIntensity()function, which employs polynomial fitting to minimize the difference between the fitted polynomial and the original data. The baseline is then removed using the Sine Model-based Iterative Peak-clipping (SNIP) method, which iteratively removes non-baseline peaks using a sine model approximation [removeBaseline() function]. The intensity of the spectra is calibrated using the Total Ion Current (TIC) method, which normalizes the intensities and allows estimation of absolute peak intensity [calibrateIntensity() function]. The estimateNoise()function visually inspects and compares noise and signal lines to determine the signal-to-noise ratio (SNR) in different elements and regions of the spectra, facilitating noise analysis.

The subsequent steps involve peak detection and analysis in all technical replicates. The detectPeaks() function applies the SuperSmoother algorithm, which uses a moving average of the second derivative to identify peaks. The parameters halfWindowSize and SNR control the peak detection windows width and the minimum required SNR, respectively. The monoisotopicPeaks() function identifies the monoisotopic peak among the detected peaks. This function takes several arguments, including minCor (minimum correlation coefficient between isotopic peaks), tolerance (maximum allowed mass difference between isotopic peaks), distance (expected distance between isotopic peaks), and size (size range of the isotopic peaks to consider). For example, a minCor threshold of 0.95 was most commonly used in this study for monoisotopic peak detection, which was determined after conducting a sensitivity analysis used in optimizing the detection of peaks while minimizing the occurrence of false positives and false negatives. A higher minCor ensures stringent correlation requirements between peaks within isotopic clusters. Lowering the minCor threshold (e.g. 0.94, 0.92, or 0.90) is advantageous to capture weaker peaks, deamidated peaks, and when the data exhibits higher levels of noise. The ‘README’ file for pre-processing provides guidance on selecting optimal parameters. To assess the number of peaks and clustering quality, we employ histograms for peak visualization. Once the peaks are identified, they are visualized using histograms to help determine the number of peaks present and the quality of clustering, subsequently removing low-quality spectra from the technical replicates. Only the subset of high-quality peaks identified here is selected for further pre-processing analysis.

In order to average the technical replicates across samples, a unique identifier is assigned to each triplicate spectrum of the same sample. Averaging the replicates reduces the Poisson noise in the mass spectra, resulting in a more accurate estimate of the true underlying spectrum and improved peak detection sensitivity. The averageMassSpectra() function (mean method) is used to compute the average of the triplicate high-quality peaks by summing up the intensities at each atomic mass unit (Da) and dividing the sum by the number of spectra in the group. The peak picking and deisotoping steps are then repeated. Finally, the pre-processed peaks are exported in .csv format and moved to the next stage of taxonomic identification in Python.

#### 2.3.2 Automated identification of species, family, and order

The algorithm was developed using Python programming language in Jupyter notebook. The codes for the semi-automated analysis are available on GitHub (https://github.com/mesve/SpecieScan), and the schematic representation of the pipeline can be seen in Fig. 1.

**Figure 2.**
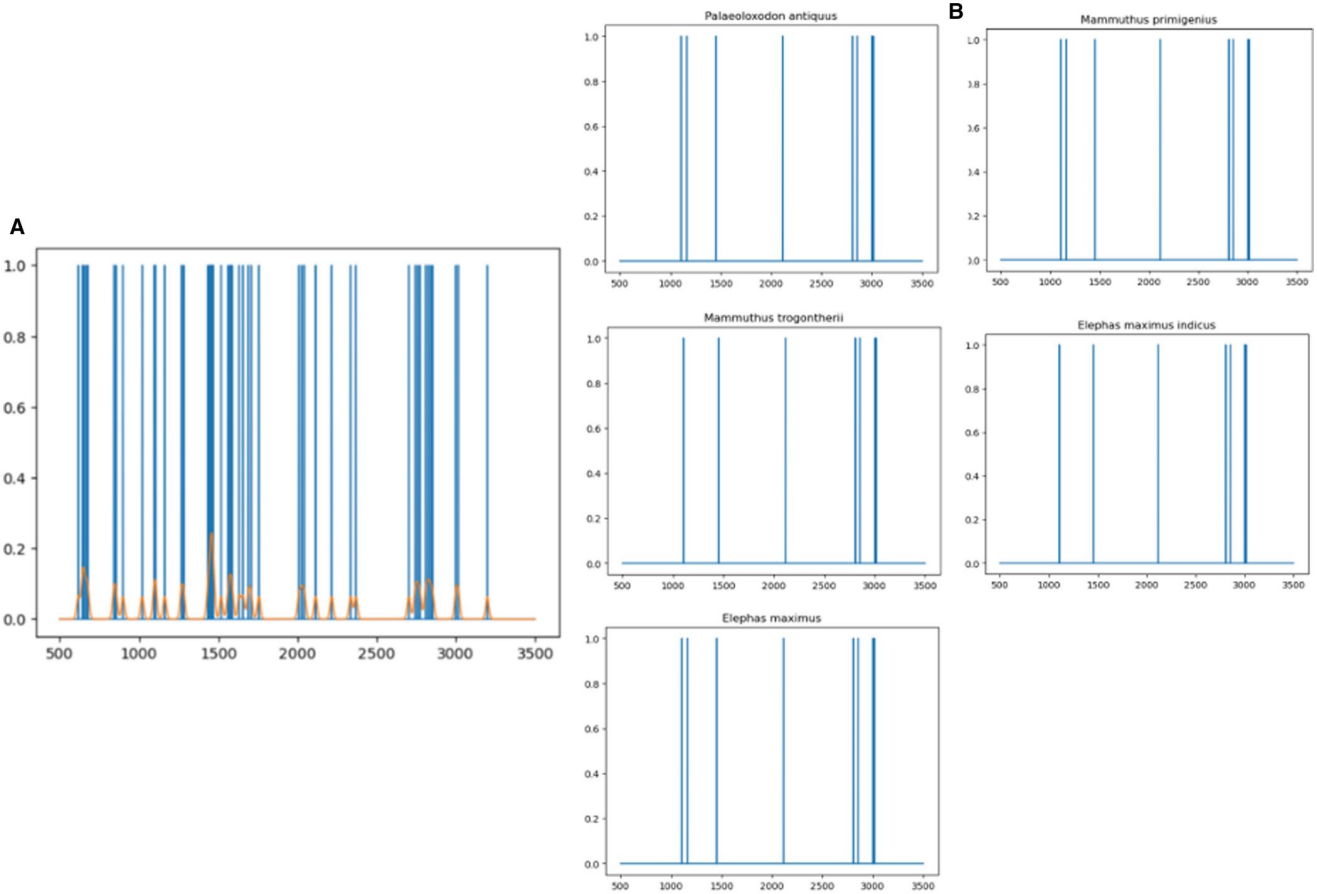
Examples of the graphs plotted by SpecieScan with the correlation scores (*y*-axis) and *m/z* values (*x*-axis). (A) An unknown sample’s mass spectrum is superimposed by all peaks from the reference database that align with peaks present in the unknown sample. (B) The matched peaks between the unknown sample and the reference database peaks. These graphs are produced after the correlation function is applied and show the peaks present in the unknown sample which align with the most likely species in the reference database. Matched species name is shown above the graph.

First, the averaged spectra from an unknown sample and the corresponding geographically specific reference database are stored in a data frame. All intensities (*y*-axis) of the sample are set to binary because the averaged spectra only include the peaks selected for analysis after pre-processing in R. A NumPy array is created, with the intensity values (*y*-axis) array set to 1 if the corresponding Da (*x*-axis) values fall within the range of −0.3 to +1.3 units (Da) away from the reference database's peaks. The ±0.3 Da represents the MALDI-ToF-MS accuracy interval, while the +1 accounts for the maximum shift in the isotope distribution of the peptides due to deamidation (cf. Welker *et al.* 2017). This wide mass tolerance range (−0.3 to +1.3) was applied to all peptide markers irrespective of their deamidation potential. Manual identification using mMass in this study revealed small occasional mass shifts larger than the restrictive ±0.3 Da, even for peptides lacking Q or N. This is likely attributed to residues contributing to deamidation, leading to the convolution of deamidated and non-deamidated peptides. Additionally, variability in MALDI-ToF-MS set-ups across different laboratories could contribute to these occasional deviations. The less restrictive mass range accommodated both deamidation and other sources of variation (van Doorn *et al.* 2012, Wilson, *et al.* 2012, Brown *et al.* 2021). The gaussian_filter1d function smooths the sample peaks, and Matplotlib is used to visualize the results (Fig. 2A). The corrcoef Numpy function calculates the correlation between the reference database's peaks and the smoothed unknown sample arrays using the ‘Species’ column of the reference database. The correlation between two arrays or matrices is a measure of the strength and direction of the linear relationship between the two sets of data (1= strong positive correlation and −1 = strong negative relationship). The resulting correlations are displayed using a series of graphs (Fig. 2B).

The intersect1d NumPy function is used to determine the common values and their indices in two arrays. Finally, each mass spectrum is scanned for known contaminants (Keller *et al.* 2008) capturing their peak locations and the names of any identified contaminants. Two types of .csv files are generated: (i) a file containing the first n species and ZooMS taxon, family, and order, their corresponding peptide markers and values common to the unknown samples and the most similar species, including the correlation scores; and (ii) a file of contaminants present in the sample. The code incorporates batch processing and automatic exporting of selected data frames into .csv files for fast processing and convenience.

Fails are automatically identified by SpecieScan when no quality peaks are present for any peptide marker. Fails were removed after SpecieScan analysis from both internal and external datasets in order to compare the manual and algorithm-produced results. Samples were considered a ‘fail’ when no ZooMS taxon was assigned to them by the original manual analyst or if no high-quality peptide markers were identified in a sample’s spectrum by the algorithm. The removal of failed samples in the statistical analysis was necessary because the algorithm often provided a possible closest match species even if only a few matching peptide markers were present. This is because SpecieScan is designed to identify the closest matches from the reference database. However, in instances where only a few matching peptide markers are present, the final output displays minimal or no peptide marker peaks with very low (near 0) correlation scores, making it evident to the user that the match is artificial. This approach is especially useful when the available spectra include incomplete marker data and helps to make educated guesses about species even when no complete combination of markers is available, which is often the case in archaeological bone fragments. However, for the purpose of comparing different identification approaches, in this study we analyse using SpecieScan a total of 557 internal and 105 external unique samples (mass spectra of averaged technical replicates).

#### 2.3.3 Statistical analysis

After calculating the correlation between an unknown sample and the reference database, a decision threshold is used to classify the unknown sample into a species. To evaluate the efficacy of SpecieScan in comparison to manual identifications, a confusion matrix was used, comparing predicted identifications with manual ones to gauge accuracy. The accuracy and robustness of SpecieScan were contingent upon the accuracy of the reference database’s peptide marker series and species alignment, as well as upon the correctness of the manual identifications of both internal and external datasets. Weighted average accuracy was computed, accounting for class imbalance in sample sizes per site. Independent t-tests were conducted on correlation scores associated with true and false ZooMS taxa identifications to determine statistically significant differences. All statistical analyses were performed using the Python programming language within a Jupyter notebook environment.

## 3 Results

SpecieScan’s accuracy in assigning identifications was on average 90.33% for ‘ZooMS taxon’, 91.84% for family, and 96.81% for order when all data (both internal and external datasets) were considered (Table 1). When we compate only manually identified datasets to SpecieScan (i.e. exclusion of Vogelherd assemblage), the accuracy achieved is 91.90% on taxon level, 93.99% for family level, and 98.29% on the order level.

**Table 1.** Performance of the SpecieScan algorithm compared to manual taxonomic identification in the different bone assemblages.^a^

	Location	Data source	*N*	Accuracy (%)		
				ZooMS taxon	Family	Order
Internal	Denisova Cave, Russia	Vienna, prepared by E.V.	290	96.21	96.9	97.59
	Vogelherd, Germany	Vienna, prepared by Bloß (2023)	267	88.01	88.76	91.38
	Internal total (weighted)		557	92.28	93.01	94.62
External	Zambia	Janzen *et al.* (2021)	73	72.6	86.3	98.63
	Australia	Peters *et al.* (2021)	24	95.83	100	100
	Azerbaijan	Wordsworth *et al.* (2021)	8	100	100	100
	External total (weighted)		105	80	90.48	99.05
Total (internal & external)			662	90.33	91.84	96.81

a The table presents the performance of the SpecieScan algorithm in comparison to the results obtained from manual taxonomic identification. The accuracy of the algorithm is measured when any species is accepted as a match [Accuracy (%) column]. For example, if the algorithm identifies *Cervus*/*Saiga*/*Gazella*/*Rangifer *sp. as the ZooMS taxa, but only *Cervus*/*Saiga*/*Gazella* sp. is identified manually, this would be considered a match.

The identification of contaminants was performed using the contaminant detection batch-processing function of SpecieScan. Aside from the intentionally introduced porcine trypsin and 4-HCCA cluster peaks, the presence of human keratin contamination was detected in all internal and external assemblages. The range in abundance spanned from relatively modest levels (19.12% of samples in the Zambian material, Janzen *et al.* 2022) to more pronounced instances (62.5% of samples in the Azerbaijani material; Wordsworth *et al.* 2021). Additionally, non-human keratins were also detected in all assemblages.

### 3.1 Internal samples

**Figure 3.**
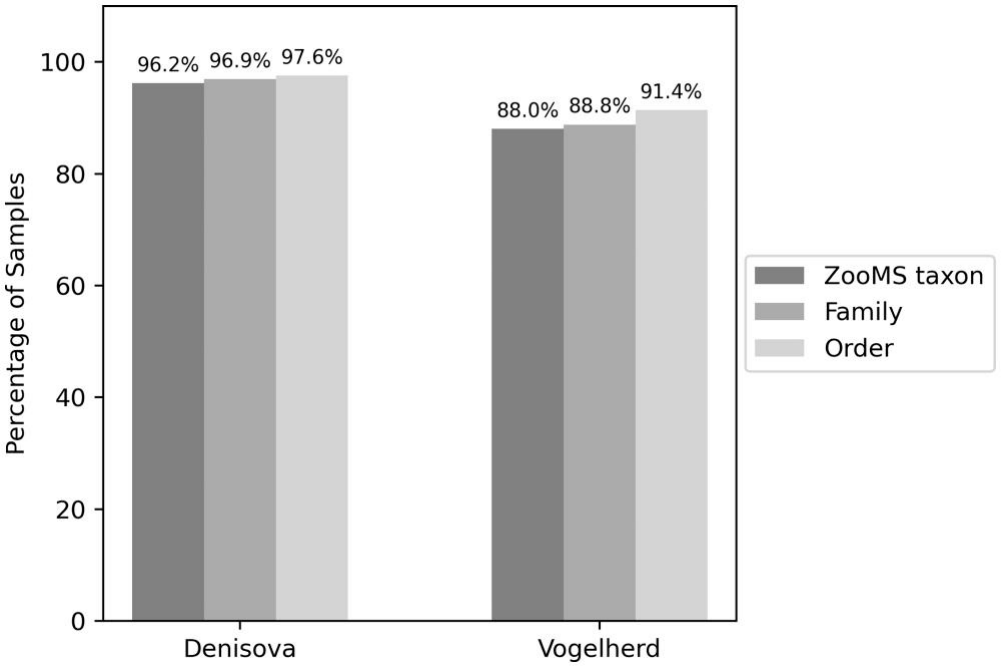
The percentage of matches of the internal samples (prepared at the Douka Lab, University of Vienna) of ZooMS taxon, family, and order between the manual and algorithm-generated labels when any correct species are accepted as a match.

The performance of SpecieScan in the identification of ZooMS taxon, family, and order was compared to the manually analysed spectra and is visualized in Fig. 3. The results demonstrate high accuracy in taxonomic identification across the analysed datasets. When considering samples prepared and analysed internally from locations including Denisova Cave, the algorithm achieved a total weighted accuracy of 96.21% for ZooMS taxa, 96.90% for family, and 97.59% for order identification. These accuracy rates indicate the algorithm's ability to effectively identify taxa based on MALDI-ToF-MS data. When the Vogelherd material is included, prepared internally by a different researcher (Bloß 2023), identified with QuickID and then checked manually , the overall accuracy was >88% for ZooMS taxa, 88.76% for family, and 91.38% for order identification (Table 1). This is primarily attributed to the limited presence of high-quality peptide markers in these samples, with an average of 4.27 markers identified. The lack of a large number of peptide markers for reliable identification was often discernible through correlation scores. Specifically, where the 75th percentile of correlation scores stood at 0.23, samples exhibited an average of 5.18 peak markers, indicating this limitation.

**Figure 4.**
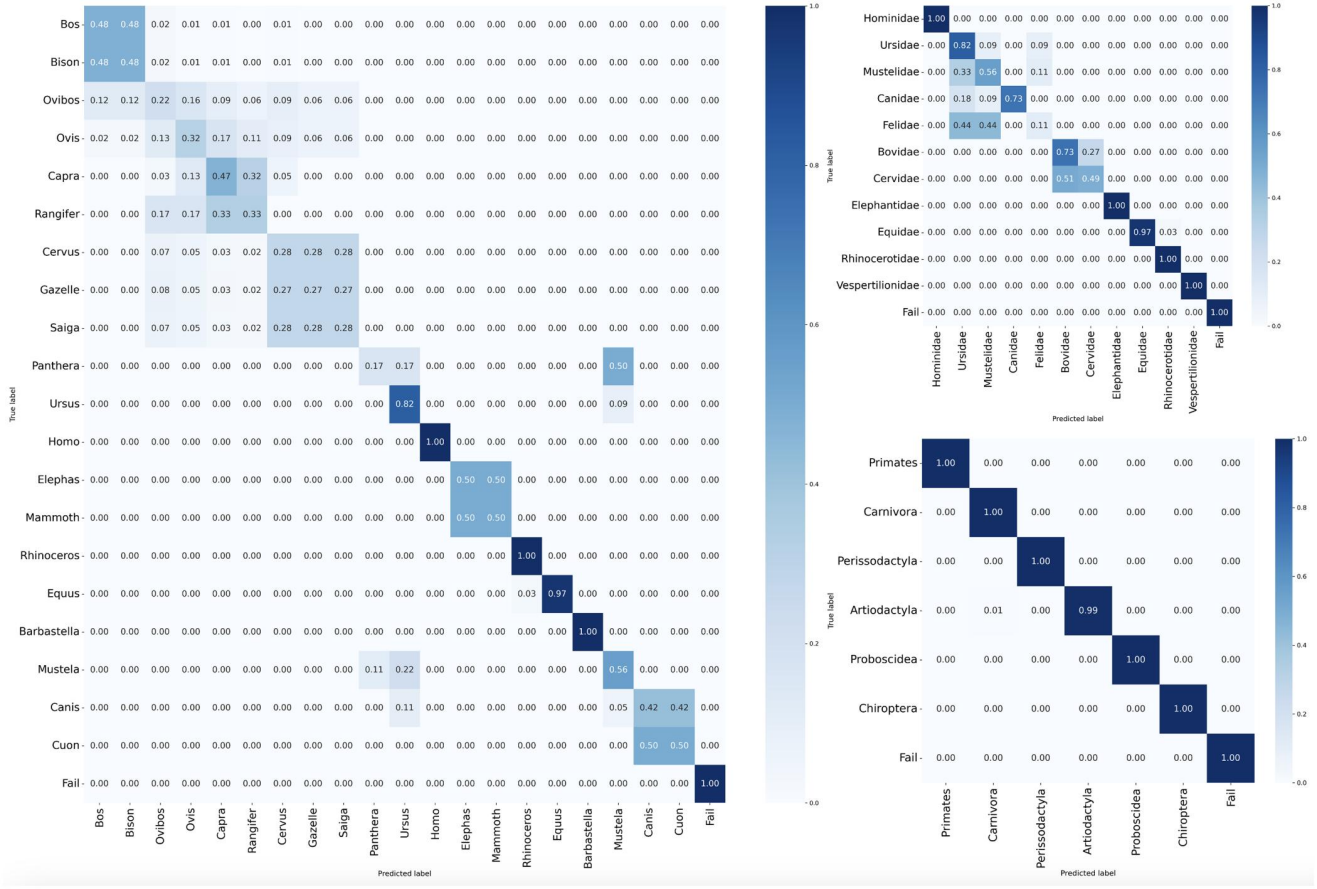
Set of three normalized confusion maps evaluating SpecieScan performance on internal samples. Each map illustrates the algorithm’s automated identification (predicted labels, *x*-axis) in comparison with manually assigned IDs (true labels, *y*-axis), highlighting the algorithm’s accuracy, and showing the areas of misclassification. Higher proportions indicate a greater number of exact matches between the predicted and true taxa. Taxon (left), family (upper right), and order (lower right): The cells in the matrix show the proportion of samples correctly classified into various taxa/family/order, highlighting the true positives, true negatives, and false positives.

Confusion matrices were utilized to identify the species with the most common misidentifications (Fig. 4). The ZooMS taxa which were most correctly identified are *Homo* sp., *Rhinoceros* sp., and *Lepus/Oryctolagus*. In the Denisova assemblage, *Ovis* sp. was the most commonly misclassified taxon, three times misidentified as *Bos/Bison* (0.34%) or *Cervus/Saiga/Gazella* (1.03%), followed by *Canis/Vulpes* as *Ursus* (0.69%), and *Equus* sp. as *Cervus/Saiga/Gazella* (1.03%). In the Vogelherd material, *Mammoth/Elephas* was the taxon misclassified most frequently (2.62%), which in all cases was due to too few peak markers preserved. Within the confusion matrix, there were additional instances of false positives, including cases of confusion between closely related species such as *Panthera* sp. and *Mustela* sp. These share similar peptide markers and from the investigated markers, differ in only one collagen peptide marker peak (COL1ɑ2 978–990 and COL1ɑ2 978–990 + 16), which is often missing from the analysed samples. Consequently, the algorithm's ability to correctly identify these closely related species is challenging due to the limited availability or complete absence of this specific peptide marker. Taxa that are not often possible to separate using ZooMS, such as *Cervus/Saiga/Gazella*, are also present in the confusion matrix (Fig. 4) but are not considered misidentification by the algorithm.

**Figure 5.**
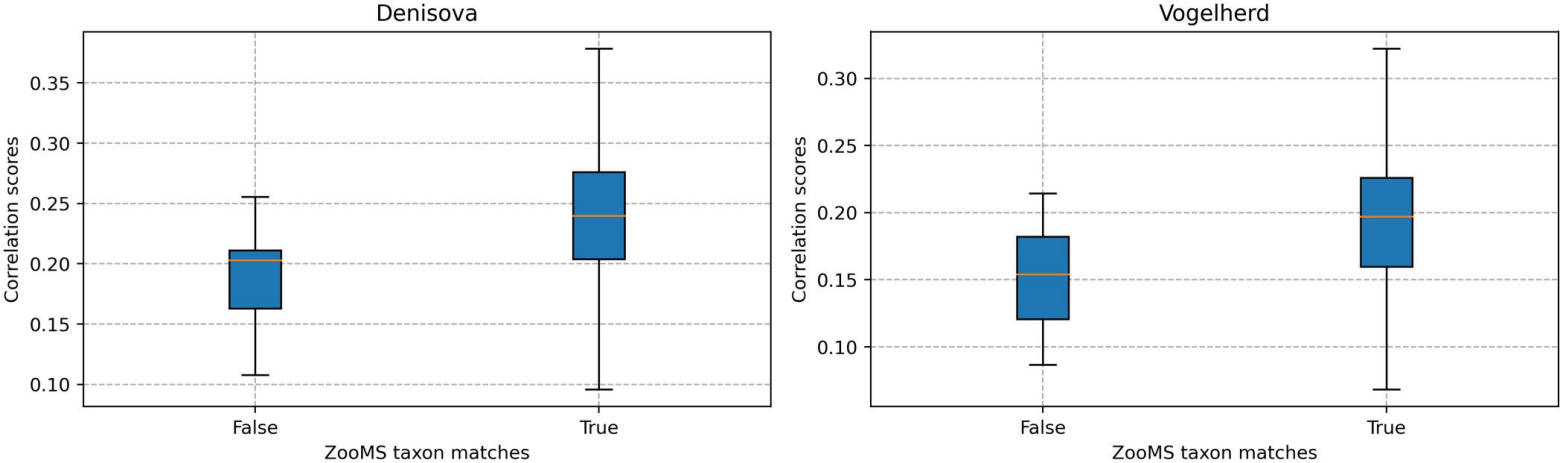
The correlation scores of the correctly identified and falsely identified samples (manually identified failed samples were excluded) in the internally produced dataset.

The analysis of the correlation scores was conducted for both the true and false label categories of the internal datasets (Fig. 5). In Denisova, the mean correlation score for the true ZooMS taxon was 0.234 ± 0.092 SD and 0.177 ± 0.049 SD for the false ZooMS taxon results. High correlation scores (>0.200) in the false negative results were due to the ∼3017.4 m/z shared among bovids in three samples (*Ovis* sp. and *Bos/Bison* (DC15019, corr = 0.256) *Ovis* and *Cervus/Saiga/Gazella* (DC15061, corr = 0.219; DC15053, corr = 0.203)). When looking at all internal samples, on average, there was no significant difference between the mean or median true and false identification correlation scores (Fig. 5).

### 3.2 External data

**Figure 6.**
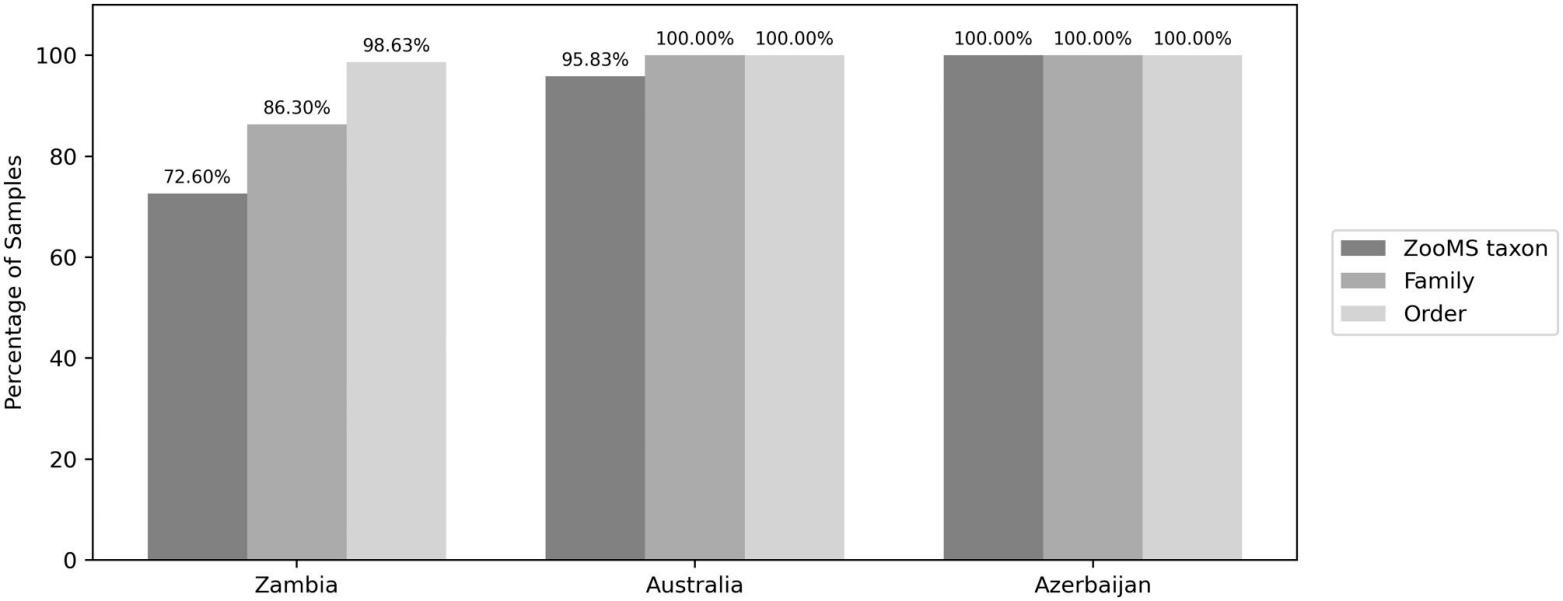
The accuracy of the SpecieScan generated IDs compared to the manually identified samples on the external dataset (Zambia, Australia, and Azerbaijan).

The analysis of external spectra using SpecieScan revealed varying levels of accuracy at the ZooMS taxon level, and this was dependent on collagen preservation and database taxonomic resolution (Fig. 6; Table 1). SpecieScan accurately identified all samples from Azerbaijan (Wordsworth *et al.* 2021), which were comprised of only two species (*Bubalus bubalis* and *Bos* sp.), both present in our current reference database. Correlation scores in this assemblage were between 0.125 and 0.388. The Australian dataset demonstrated 95.83% accuracy on the ZooMS taxon level when juxtaposed with manual identifications outlined in (Peters *et al.* 2021). The only discrepancy was observed in sample CP955, which had only two peptide markers preserved (COL1ɑ2 484–498 and COL1ɑ2 757–789). Consequently, the reliability of the provided ID for this sample appears questionable, irrespective of it being identified manually or by SpecieScan. In fact, the Australian assemblage was so poorly preserved that only eight samples retained four or more peptide markers, a usual cut-off for proclaiming a sample identifiable or ‘failed’.

**Figure 7.**
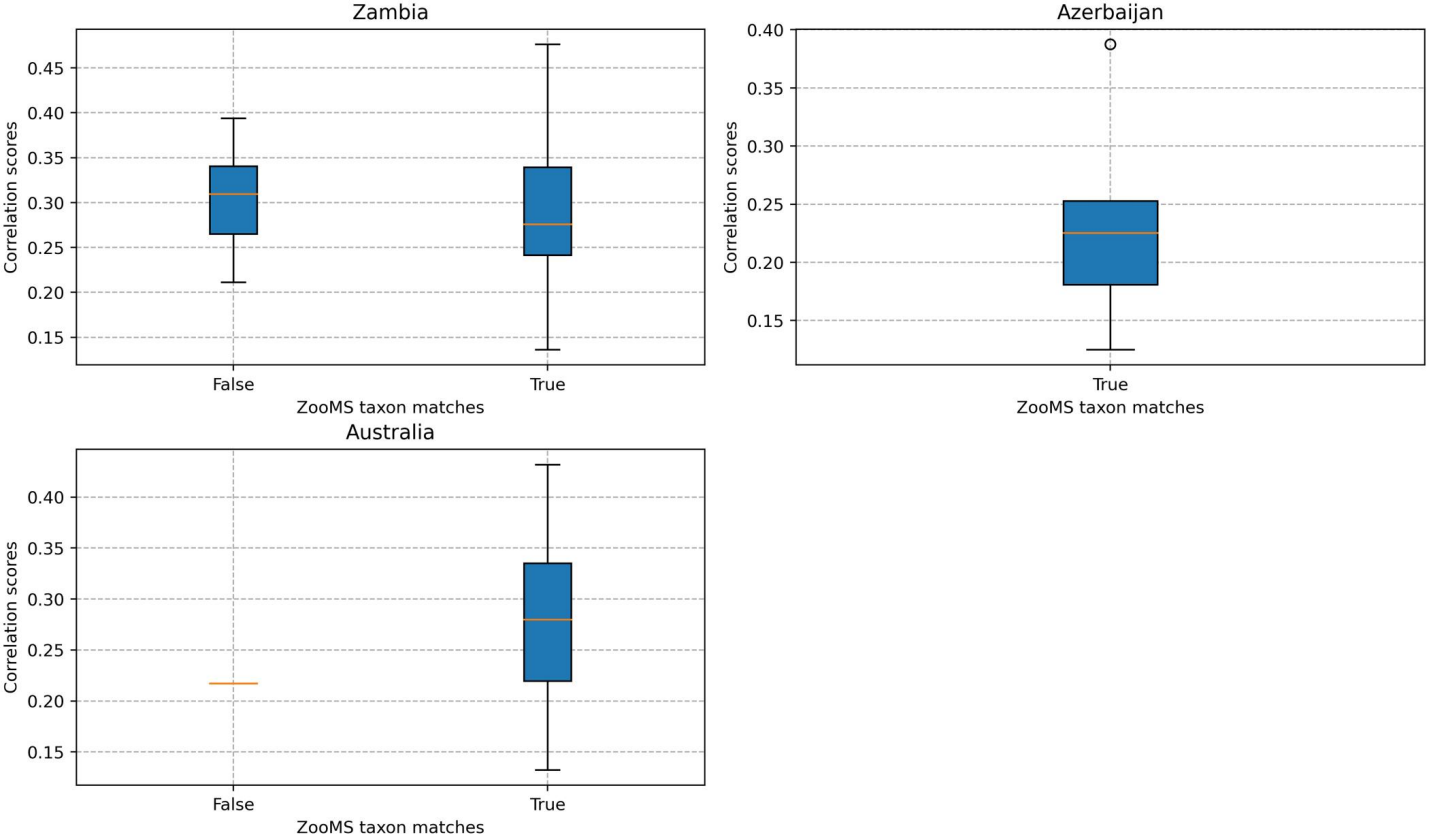
The correlation scores of the correctly identified and falsely identified samples (manually identified failed samples were excluded) in the externally produced spectra.

The algorithm was the least accurate when analysing spectra from Janzen *et al.* (2021). The most common mistakes in this dataset involved the misclassification of different species of bovids (15%). Additionally, some manually identified bovids were misclassified as *Giraffa* sp. (8.2%), while an *Orycteropus* sp. specimen was identified as *Rattus* sp. (1.36%). Occasionally, SpecieScan was able to identify or narrow down the ZooMS taxon more than the manual analysis in the original paper (Janzen *et al.* 2021). For example, sample KAV458 (hypsodont/cheektooth) was not identified with manual ZooMS analysis in Janzen *et al.* (2021), and the ZooMS ID given to this sample was ‘Unknown. Dog?’ by the authors. However, SpecieScan identified this sample as *Loxodonta* sp., a species of *Elephantidae* with a large population in Zambia (Table 2).

**Table 2.** Results of taxonomic identification for sample KAV 458 (Janzen *et al.* 2021) using manual analysis and SpecieScan.^a^

Identification	Peaks (Da)	ZooMS ID	Family	Order	Correlation
Janzen *et al.* (2021)		‘Unknown. Dog?’			N/A
Manual	1105.6, 1178.5, 1453.7, 1580.8, 2115.1, 2809.1, 2853.3	*Loxodonta* sp.	*Elephantidae*	*Proboscidea*	N/A
SpecieScan	1105.6, 1453.7, 1579.8, 2115.1, 2808.4, 2853.4	*Loxodonta* sp.	*Elephantidae*	*Proboscidea*	0.235

a In the original study (Janzen *et al.* 2021), the ZooMS taxonomic identification was recorded as ‘Unknown. Dog?’, SpecieScan identified it as Loxodonta sp. Note that the disparities in the precise peak locations between the manual analysis and SpecieScan analysis are attributed to the ±0.3 Da confidence interval of the instrument. Additionally, the presence of a + 1 variation is due to the maximum shift caused by peptide deamidation and other variables. Note, while the manual analysis indicated the presence of multiple species within the Elephantidae family, only one species, namely *Loxodonta* sp., is known to exist in Africa. This sample was mistakenly assigned as ‘unknown/dog’ in the published version of the dataset and was indeed identified as *Loxodonta* sp. (Janzen, personal communication).

In terms of the correlation scores of the external samples (Fig. 7), there was notable similarity between the true and false scores (mean correlation score for ZooMS-based ‘true’ identifications = 0.282 ± 0.09 SD; ‘false’ identifications mean = 0.305 ± 0.05 SD). However, many of the ZooMS-based identifications manually analysed in Janzen *et al.* (2021) belonged to several bovid species separated by only one or two peptides, making their true separation challenging. SpecieScan occasionally assigned relatively high correlation scores to these specific bovids. The minimum number of peptide markers needed to be present for consistently correct identification was four in the Australian assemblage and any number of peptide markers in the Azerbaijani assemblage.

## 4 Discussion

In this study, we establish a pipeline for semi-automated taxonomic identification of MALDI-ToF collagen peptide mass spectra. Instead of following a specific order of peptide markers, the spectral peaks are viewed comprehensively and matched to the reference database’s peptide markers. Manual analysis is labour-intensive and sometimes challenging due to the occasional absence of one of the primary peptide peaks in the established sequence of peak analysis. For example, in the case of sample DC15240 from Denisova Cave, following the application of filtering for COL1ɑ2 484–498, none of the potential COL1ɑ2 793–816 peaks were detected, thereby precluding the possibility of obtaining a match in the reference database. It was only through the manual experimentation with alternative marker filtering orders that this specimen was eventually identified as belonging to an *Equus* sp., despite the absence of the COL1ɑ2 793–816 peak. Similarly, in multiple samples derived from *Rhinoceros* sp., such as DC15121, we consistently observed that peaks corresponding to COL1ɑ2 454–483 at approximately 2792 Da were regularly present. Filtering for this peak marker in the reference database results in the misidentification of the sample as South American tapir, a species that is inappropriate for the Eurasian geographical context. When such scenarios occur, the constraints imposed by the reference database’s filtering mechanisms hinder the exploration of alternative Da values associated with the other peak markers.

SpecieScan performs especially well when collagen is well preserved, such as the majority of bone fragments we analysed from Denisova Cave, and when the reference database for the geographical region has relatively high resolution, such as in northern Eurasia. It is efficient, consistent, and saves time compared to manual analysis, effectively overcoming the issue of missing occasionally prominent peaks (e.g. COL1ɑ2 793–816). In manual analysis, the presence or absence of certain peptides can lead to incorrect identification due to the way filtering functions in the reference database. We demonstrate here that SpecieScan can overcome these problems due to its underlying mechanism of viewing the spectra peaks as a whole without ordered filtering and by using geographically relevant reference databases.

The correlation scores produced by SpecieScan quantify the degree of alignment of an unknown sample’s mass spectra with known species’ spectra. Correlation scores in SpecieScan are inherently relative, thus establishing a universal threshold for these scores across diverse locations is challenging. Our study revealed varying maximum correlation scores even among true identifications across different sites. Scores are influenced by inherent factors such as collagen preservation, marker availability, and species similarity. Hence, we advise interpreting these correlation scores only contextually without relying on these scores for identifications. The correlation scores should primarily aid in determining the best matching taxonomic group among the x closest fitting species selected during the automated analysis.

Some of SpecieScan’s false negative results were due to the misclassification of *Ovis* sp. This was most often due to either the COL1ɑ2 757–789 peptide containing four hydroxyprolines or a conserved bovid peptide at COL1ɑ2 757–789 (peak at around 3017.4 Da) overlapping with the ZooMS markers of *Ovis* sp., *Capra* sp., *Bos/Bison*, *Cervus/Saiga/Gazella*, and *Sus* sp. Therefore, when a peak at ∼3017 Da is present at this peptide marker and four or less peptide markers are identified, caution and manual identification is recommended without filtering the COL1ɑ2 757–789 marker.

In cases where the analysed sample yields fewer than four peptide markers, the SpecieScan algorithm might yield multiple species results with the same or comparable correlation scores. This can be attributed to the shared peptides among these species and the lack of the characteristic peptides differentiating different taxa. For example, sample DC12739 was identified as either *Mustela* or *Phocid* sp. by SpecieScan, based on three common peptide markers (1453.7, 1566.8, and 2853.4) with identical correlation scores of 0.11 for both species. In such cases, researchers should exercise common sense, taking into consideration the geographical location and time period of the sample. For example, given that this sample originates from Denisova Cave, far from large bodies of water where *Phocid* sp. might reside, *Mustela* sp. was assigned as the identified species. Therefore, when four or less peptide markers are present, we recommend manual inspection of the spectra and/or only tentative assignations; or that the sample is failed completely.

Other false negative results are attributed to poor bone preservation and contaminant peaks which are of protein origin and can be confusing in both manual analysis and when using SpecieScan. Autodigested trypsin peaks (Keller *et al.* 2008) and matrix peaks (<1000 Da) are common contaminants, but keratins from skin and clothing are the most abundant contamination in ZooMS spectra. These can occur during laboratory preparation of samples or introduced by human handling at any time in the sample’s previous history (Hendy *et al.* 2018, Richter *et al.* 2022). Keratin contaminants have been identified and reported in ancient mammalian bone (Procopio *et al.* 2021), and some keratin contamination was found in salmonid bones (Korzow Richter *et al.* 2020). Through an integrated contamination detection batch-processing function in SpecieScan, it is possible to scan for the masses (Da) associated with specific contaminants. In this work, human keratin peaks were consistently detected in both internally and externally analysed assemblages, irrespective of the individuals responsible for their preparation. Other possible contaminant peaks we detected include non-human keratin, bovine Casein a-S1, and minuscule amounts of Bovine Serum Albumin (BSA; detected in the Denisova and Zambian assemblages). Although keratin is expected in faunal bone, human keratins, BSA, and bovine casein a-S1 are most likely contaminants from laboratory processing of samples. When SpecieScan identifies masses of potential contaminants in samples, caution should be exercised when considering these peptide markers for taxon identification purposes. Our findings underscore the importance of rigorous contamination control measures in ZooMS laboratory analysis, such as rigorous cleaning of equipment and the use of face masks during sample preparation, in addition to the use of blanks in all parts of sample processing. Other factors affecting the presence of peptide marker peaks in spectra might be due to glycosylation, glycation, cross-linking, issues during the chemical preparation (incomplete digestion), poor ionization, as well as different PTMs and non-enzymatic peptide fragmentation (Richter *et al.* 2022).

There was a minor difference in the performance of SpecieScan between the internal samples prepared and analysed by two different users, from two distinct sites (Denisova Cave versus Vogelherd Cave). This difference in performance is likely due to the preservation of the material, methods of preparation and analysis, or the way species were identified manually.

When comparing ZooMS taxon-level assignations, SpecieScan performed worse for the classification of externally produced data than in the internal ones. We attribute this to collagen preservation issues pertinent to many of the external data (the samples derive from Africa and Australia), as well as the lack of high-resolution reference databases for these two continents. However, when analysing the Azerbaijani samples with the Eurasian reference database, which has the highest resolution, SpecieScan reached 100% accuracy. The accuracy of these results is contingent upon the quality of both the reference database available for the region, the preservation of collagen, and the accuracy of the manual identification.

We hope that as the algorithm becomes available, SpecieScan will be tested on more diverse samples prepared in different laboratories. Due to the lack of raw MALDI-ToF spectra in the published literature concerning archaeological material, we were only able to retrieve three datasets to test SpecieScan. While recently published literature includes freely accessible data, these are not raw data but pre-processed formats (e.g. spectra whose baseline is removed and averaged spectra from technical replicates). This precludes other researchers to use and evaluate the data and reproduce the work of other analysts. Our strong recommendation for ZooMS researchers is to aim at publishing individual replicate spectra in .mzML or .txt file format in the future.

## 5 Conclusion

We have successfully developed and implemented an easy-to-use algorithm (SpecieScan) for semi-automated taxonomic identification of MALDI-ToF-MS data obtained from bone collagen. The SpecieScan algorithm incorporates a comprehensive pre-processing workflow that includes data visualization, quality control, smoothing, baseline removal, intensity calibration, noise estimation, peak detection, automated taxonomic identification, and major contaminants scanning. The pre-processing steps are carried out in the R programming language using various packages, and the taxonomic identification was automated in Python programming language.

The SpecieScan algorithm accelerates data analysis of ZooMS spectra and establishes a correlation score for the taxonomic identifications, which has so far been impossible to obtain with manual identifications. The code can be run by researchers with minimal to no knowledge of programming and with only a basic understanding of data analysis of MALDI-ToF mass spectra.

The results obtained from the application of SpecieScan to unknown and previously published data demonstrated high accuracy and reliability in identifying ZooMS taxa at rates of successful identification as high as 96.21%–100% in well-preserved material (e.g. Denisova Cave and Azerbaijan) when compared to manually identified ZooMS taxa.

Despite acknowledged limitations, the newly developed SpecieScan algorithm offers an efficient and reliable approach for the taxonomic identification of MALDI-ToF-MS data for ZooMS purposes when compared to manual analysis. Future research can focus on comparisons between the algorithm by Hickinbotham *et al.* (2020) and SpecieScan regarding performance and required input, and further refinements of the algorithm by incorporating additional pre-processing techniques and optimizing the peak detection and identification steps. Expanding reference databases and more rigorous validation studies with a larger and more diverse set of samples from various geographic regions and taxonomic groups, would consolidate the algorithm's robustness and applicability.

## Data Availability

The SpecieScan algorithm, the original raw data, reference databases, results sheets, and detected contaminations generated in this study are available on GitHub at https://github.com/mesve/SpecieScan (doi: 10.5281/zenodo.8055426).
